# The implication of ciliary signaling pathways for epithelial–mesenchymal transition

**DOI:** 10.1007/s11010-023-04817-w

**Published:** 2023-07-25

**Authors:** Bang-Hua Zhong, Ming Dong

**Affiliations:** Department of Gastrointestinal Surgery, The First Hospital of China Medical University, Shenyang, Liaoning China

**Keywords:** Primary cilium, Signaling pathway, EMT, Ciliogenesis, Cancer

## Abstract

Epithelial-to-mesenchymal transition (EMT), which plays an essential role in development, tissue repair and fibrosis, and cancer progression, is a reversible cellular program that converts epithelial cells to mesenchymal cell states characterized by motility-invasive properties. The mostly signaling pathways that initiated and controlled the EMT program are regulated by a solitary, non-motile organelle named primary cilium. Acting as a signaling nexus, primary cilium dynamically concentrates signaling molecules to respond to extracellular cues. Recent research has provided direct evidence of connection between EMT and primary ciliogenesis in multiple contexts, but the mechanistic understanding of this relationship is complicated and still undergoing. In this review, we describe the current knowledge about the ciliary signaling pathways involved in EMT and list the direct evidence that shows the link between them, trying to figure out the intricate relationship between EMT and primary ciliogenesis, which may aid the future development of primary cilium as a novel therapeutic approach targeted to EMT.

## Introduction

The epithelial-to-mesenchymal transition (EMT) is a reversible cellular program that converts epithelial cells to mesenchymal cell states characterized by the motility-invasive properties with altered expression of the epithelial marker E-cadherin and the mesenchymal marker vimentin [1]. The classified EMT programs as EMT-type 1, 2 and 3 by biological context operate vital roles for gastrulation, neural crest delamination during embryonic development [2], tissue repair and fibrosis [3], cancer progression [4]. Although it has been viewed as a binary process between these two extremes, recent studies indicated that EMT is a continuous dynamic transition process from epithelial to completely mesenchymal states. It passes through intermediate hybrid states that express different levels of epithelial and mesenchymal markers and exhibits intermediate morphological, transcriptional and epigenetic features [5, 6].

EMT is mainly regulated by a core set of EMT-activating transcription factors (EMT-TFs), including SNAIL (SNAI1) and SLUG (SNAI2), the basic helix–loop–helix factors TWIST1 (TWIST) and TWIST2 and the zinc finger E-Box binding homeobox factors ZEB1 and ZEB2 to repress epithelial genes like the E-cadherin and upregulate the expression of mesenchymal genes like vimentin [7]. Meanwhile, multiple signaling pathways such as transforming growth factor-β(TGFβ), WNT, NOTCH, and phosphatidylinositol 3-kinase-AKT kinase (PI3K-AKT) cooperate in the initiation and progression of EMT by ultimately inducing the expression of various EMT-TFs during normal development, wound healing and carcinoma progression [8].

The primary cilium is a solitary, hair-like organelle elongating from the cell surface into extracellular environments in almost all human cell types [9]. As a functional antenna, besides its role to receive information from the environment and locally transduce it into a cellular response, researchers also revealed that primary cilium could transmit signals through released extracellular vesicles (EVs) [10, 11]. Thus a suite of soluble ligands such as Sonic hedgehog (SHH), Receptor Tyrosine Kinases (RTK), WNT, G-protein-coupled receptors (GPCR) and NOTCH were dynamically received and transduced in part or sum by primary cilium [12], which activates intracellular or extracellular signaling pathways to regulate cell polarization, differentiation, proliferation, specific immune cell functions and metabolism [13, 14]. The dysfunction of primary cilium was also proposed as a prerequisite step of cancer development and stem cell identity [15, 16]. Although the study on role of primary cilium in cancer is still undergoing, it is well accepted that the primary cilium appear to have a dual function in different cancers or even same cancer by either promoting or blocking tumorigenesis depending on the nature of the oncogenic initiating event[17].

Emerging evidence indicated that primary cilium mutation interrupted EMT in multi organs and tissues, including epicardial tissue, kidney epithelial cells, pancreatic β-cells and the retinal pigment epithelium (RPE) [18–22]. Recent research also revealed the connection between EMT and primary ciliogenesis in the context of cancer such as glioblastoma(GBM) and triple-negative breast cancer (TNBC) [23, 24], bladder cancer [25], proposing the relevance of primary cilium as a potential target in cancer EMT. Nevertheless, the mechanism is still complicated. The focus of this review is to described the current knowledge about the influence of ciliary signaling pathways for EMT and try to figure out the intricate relationship between EMT and primary ciliogenesis, which maybe aid the future development of primary cilium as a novel therapeutic approach targeted to EMT.

## Ciliary signaling pathway involved in EMT

EMT can be induced by several intracellular signaling pathways that are known to induce the expression of various EMT-TFs [8, 26],in which the primary cilium function as a mediator by detecting signals from the extracellular environment and displaying specific receptors required for signal interception and the downstream molecular effectors [27]. In this section, we focus on the ciliary signaling pathways functioned in EMT including TGFβ, HH, WNTs, NOCTH and RTK.

### Primary cilium-based TGFβ signaling

The superfamily of TGFβ comprises more than 30 different ligands of TGFβ/activin/Nodal and bone morphogenetic protein (BMP) subfamilies to activate serine/threonine kinases receptors of types I and II (TGF-βRI/II and BMP-RI/II, respectively) in paracrine or autocrine manners [28], which function in a multitude of cellular processes during development and in the maintenance of tissue homeostasis in the adult.

Increasing evidence demonstrate the remarkable role for the primary cilium in regulating TGFβ signaling. The first report of active TGFβ signaling in the primary cilium was based on studies using cultured mice and human fibroblasts. The localization of TGFβ receptors were identified in the tip of the cilium using immunofluorescence microscopy, when activated the TGFβ receptors migrated to the ciliary base to phosphorylate SMAD2/3 and ERK1/2. TGFβ signaling were reduced at stunted primary cilium in Tg737orpk fibroblasts, indicating that the primary cilium regulate the output of canonical and non-canonical TGFβ signaling to control varied cellular responses [29]. Further studies supported the idea that primary cilium is vital for balancing the output of TGF-β/BMP signaling to control varied cellular responses such as mice heart development [30], migration of human bone mesenchymal stem cells [31] and migration and tumor metastasis in mammary cancer cells [32]. TGFβ-1 signaling and SMAD3 activation were impaired in ciliated fibroblasts which are enriched in areas of myocardial injury upon primary cilium being removed, extracellular matrix protein levels and contractile function were also impaired, suggesting a pivotal role of primary cilium in disease-related pathological cardiac remodeling via regulating TGF-β1/SMAD3 axis [33]. Meanwhile, IFT80 deletion down-regulated the TGF-β signaling pathway by inhibiting the expression of TGF-βI, TGF-βR, and phosphorylation of Smad2/3 in the fracture healing [34]. Moreover, other proteins that regulate receptor transport and feedback inhibition mechanisms are enriched in cilia-centrosome axis, which underlined the importance of cilium in coordinating TGFβ signaling. For instance, loss of CEP128, a sub distal appendage protein which recruited RAB11 [35] that is responsible for endosomal recycling of TGFβ receptors to cilium, impaired phosphorylation of SMADs leading to defective organ development in zebrafish [36] and in male fertility [37]. Interestingly, TGF-β treatment resulted in suppresses average length of cilia by upregulating histone deacetylase (HDAC) activity [38] and regulating Ift88 gene expression at least in part via posttranscriptional manner [39].Conversely, SMAD7, the feedback inhibitor of TGFβ signaling, which localize to ciliary base and restrain excessive signaling from primary cilium [29] had been proposed to limit ARL6-mediated ciliary localization of TGFβ receptors and suppress tumor cell migration and invasion by restricting cross-talk between TGFβ receptors and SMO in HH signaling [32].

### Primary cilium-based Hedgehog signaling

Hedgehog (HH) signaling is an intricate, highly conserved evolutionary pathway that plays an instructional role during embryonic development, stem cell biology and tissue homeostasis [40].

Primary cilium play a vital role in HH signal transduction was well established. At first, researchers found that defects in intraflagellar transport (IFT) disrupted sonic hedgehog (SHH) signaling based on the neural tube patterning of mice embryos [41]. Further evidence indicated that dysfunction of primary cilium might disturb HH signaling in different models. For example, The ADP ribosylation factor-like GTPase 13B (ARL13B) is extensively used as a primary cilium marker and was found to mediate ciliary entry of SMO, mice lacking ARL13B had abnormal SHH signaling [42, 43]. It is well accepted that in vertebrates, the core HH machinery components, including Patched 1 (PTCH1), GPR161 and Smoothened (SMO), suppressor of fused (SUFU) and the glioma-associated oncogene (GLI) transcription factors which dynamically localized to primary cilium throughout the cell cycle [44, 45].To be more specific, when signaling is activated, in addition to PTCH1 removal, SMO accumulates in the ciliary membrane [46] and changes the processing of Gli, giving rise to the Gli variant GliA, which then leaves cilium and enters nucleus to activate downstream genes that change the differentiation program [47]. At the same time, GPR161, which was identified as a negative regulator of SHH signaling [48], was removed from primary cilium [49], in a β-arrestin-dependent manner [50].Therefore, canonical HH signaling is strictly dependent on intact primary cilium and IFT [40]. Prominin-1 (Prom1, also known as CD133),which involve in the maintenance of ciliary structure and function [51], mediate the transduction of Glis2 from primary cilium to nucleus and activation of the direct downstream target STAT3 in mouse incisor cervical loop epithelium-associated stem cells [52].The role of primary cilium in regulating non-canonical HH pathways is still little known, but ciliary protein IFT80 can inhibits HH non-canonical signaling via HH–Smo–Gai–RhoA–stress fiber signaling while promoting HH canonical signaling in mouse osteoblast differentiation [53].

Remarkably, studies show that primary cilium can either boost or inhibit tumorigenesis depended on the underlying carcinogenic factors in cancer types [54, 55]. Ciliary ablation strongly inhibited BCC-like tumors induced by an activated form of Smoothened. In contrast, removal of cilia accelerated tumors induced by activated Gli2, a transcriptional effector of Hh signaling [54].Conversely, knocked down of Stk11, also known as Lkb1,which was identified as a HH pathway gene using genome-wide RNA interference (RNAi) screen resulted in increased Gli3R abundance and cilia disassembly [56], suggesting that HH pathway may also regulate primary ciliogenesis and maintenance in a feedback loop.

### Primary cilium-based WNT signaling

The WNT signaling pathway is a critical molecular rheostat to regulate development and adult tissue homeostasis, and its dysregulation has been found in many cancer types. For example, alterations in the WNT signaling pathway are a near-universal feature of colorectal cancer driven by truncating mutations in adenomatosis polyposis coli (APC) [57]. WNT signaling can induce β-catenin-dependent (canonical) and β-catenin-independent (non-canonical) signaling. The latter includes calcium signaling or the planar cell polarity (PCP) pathway [58].

Several core WNT pathway components were found to have ciliated localization [59, 60] and INVERSIN inhibits the canonical WNT pathway by targeting cytoplasmic disheveled (Dsh or Dvl1) for degradation first link WNT signaling with primary cilium [61]. Some studies supported that primary cilium (or cilium-associated proteins) promote tumors by restraining WNT signaling activity from regulating proliferation and differentiation. For instance, some researchers found that downregulation of cilium proteins including KIF3A, IFT88 and OFD1 in mice embryos, primary fibroblasts and embryonic stem cells leads to accumulating β-catenin, which subsequently increases the transcription of WNT target genes [62]. Further research revealed a spatial mechanism that the primary cilium diverted Jouberin (JBN), a ciliopathy protein and β-catenin-positive regulator, by facilitating its nuclear localization away from the nucleus and thereby negatively affecting WNT signaling [63]. Studies [64, 65] have shown that deletion of BBSome (a complex containing eight Bardet Biedl syndrome proteins: BBS 1, 2, 4, 5, 7, 8, 9 and 18) influenced stabilization and post-translational modification of β-catenin by perturbing proteasomal degradation and regulating histone deacetylase 6 (HDAC6), an enzyme that deacetylates β-catenin at lysine 49 and inhibit β-catenin phosphorylation at serine 45 [66], to alter the regulation of downstream WNT targets and establish the PCP mutant phenotypes, including open eyelids and disorganized stereocilium in mice model [67] and left–right asymmetry in zebrafish [68]. Meanwhile, increased phosphorylation of transcriptionally active serine 552 on β-catenin was observed in BBS8 knockdown RPE cells, offering proof for BBS8 suppressing canonical WNT signaling [69, 70]. Further research revealed that deletion of BBS8 disrupted asymmetric accumulation of the core PCP protein Vangl2 in cochlear cells, suggesting a role for BBS8 possibly upstream of core PCP asymmetry. Loss of INVERSIN and OFD1 led to PCP-regulated convergent extension defects in vertebrates also supported the involvement of cilium in non-canonical WNT signaling [61, 71]. Besides that, the deletion of BBSome had increased the release of small EVs (smEVs) loaded with WNT-related molecules and smEVs derived from ciliopathy patient renal tissues dampened the WNT response in target cells, in contrast with control tissues [72]. Although the involvement of primary cilium in WNT signaling was still contentious and undergoing, these data suggest that primary cilium is required for canonical or non-canonical WNT signaling.

### Primary cilium-based NOTCH signaling

The NOTCH signaling is an evolutionarily conserved pathway crucial in regulating a diverse array of cell fate decisions during lineage commitment, differentiation, cell cycle progression, maintenance and self-renewal of stem cells [73]. Activation of the NOTCH signaling depends on the combination of Delta-like and Jagged families NOTCH ligands with the receptors of NOTCH1-4 [74].

A role for the primary cilium in NOTCH signaling was first identified in 2011 when the knockdown of IFT proteins in both cultured keratinocytes and embryonic epidermis cells led to significant NOTCH defects, while differentiation defects were cell-autonomous and rescued by activated NOTCH [75]. Subsequent research by the same group showed that the small GTPase (ARF4)-dependent polarized exocytosis acts through the basal body-ciliary complex to regulate NOTCH signaling during epidermal differentiation spatially [76]. Loss of primary cilium in corneal epithelial diminished NOTCH activation to reduce cell proliferation, accompanied by NOCTH1 and NOTCH2 receptors which were normally expressed and nuclear NOTCH1 Intracellular Domain (NICD1) was severely reduced [77]. Hemodynamic alteration is perceived by endocardial cells through primary cilium that mediates the upregulation of hemodynamic responsive factors klf2a and klf2b. The increased klf2 gene expression activates endocardial NOTCH signaling to promote ventricle regeneration [78]. Liu et al. reported that primary cilium regulates hematopoietic stem and progenitor cell specification through NOTCH signaling in zebrafish [79]. A study by Leitch et al. showed that the absence of BBSome subunits resulted in the upregulation of NOTCH signaling in transgenic zebrafish NOTCH receptor cell lines and human cultured cells. The reduction of BBS1 or BBS4 diminished the recycling of NOTCH from early endosomes [80]. Although the exact regulatory process and the underlying mechanisms remain incomplete, these evidence depicted that primary cilium regulates NOTCH signaling by modulating the spatial localization of NOTCH signaling intermediates.

### Primary cilium-based RTK signaling 

RTK signaling consists of 58 members, which plays an essential role in various cellular processes including growth, motility, differentiation and metabolism in humans [81]. Among them, epidermal growth factor receptors (EGFRs), platelet-derived growth factor receptors (PDGFRs), insulin-like growth factor receptors (IGFRs), fibroblast growth factor receptors (FGFRs) and Tropomyosin receptor kinase B(TRKB) were linked to primary cilium by ciliary localization and associated functions [82]. PDGFRα-mediated signaling depends upon its ciliary localization in quiescent fibroblasts and fibroblasts derived from Tg737^orpk^ mutants, which failed to form normal cilium and upregulate the level of PDGFRα. Thus, the Mek1/2-Erk1/2 pathway could not be activated [83]. Further research from the same team demonstrated that in coordination with the cytoskeletal reorganization, the fibroblast primary cilium function via ciliary PDGFRα signaling to monitor directional cell migration during wound healing [84]. The depletion of cilium disassembly complex components is sufficient to induce ciliogenesis in a subset of glioma stem cells (GSCs). The process occurs via relocating PDGFRα to the newly induced cilium and reintroducing cilium switches GSCs from self-renewal to differentiation. This can prevent the infiltration of GSCs into the brain, suggesting cilium induction as an attractive strategy to intervene in GSCs proliferation via PDGFRα signaling [85]. Less activated AKT, which was shown to be activated at the ciliary base in a PDGF-AA-dependent manner [86], led to impaired migration response in BBS1^M390R/M390R^ MEF cells [87]. Mechanisms that regulate ciliary targeting of PDGFRα and balance the output of PDGF-AA-mediated signaling have been revealed. A recent study revealed that IFT20 depletion with a consequent loss of cilium-induced mislocalization of PDGFRα to the plasma membrane could impair negative feedback for pathway regulation. This is due to the destabilization and degradation of the E3 ubiquitin ligases [88]. Although PDGFRα is one of the most studied RTKs from a ciliary perspective, other RTKs have also been linked to the primary cilium in various ways, yet the significance and regulation mechanism of their ciliary localization requires further research. For example, phosphorylation and activation by brain-derived neurotrophic factor (BDNF) of its target receptor, TRKB, decreased upon loss of BBS4 expression in cultured cells, thereby linking ciliary TRKB signaling to neuronal phenotypes associated with BBS [89]. Retinal ganglion cell (RGC) primary cilium-concentrated IGF1R could be lost after the injury, reducing IGF1 potency. While regenerated RGCs relocated IGF1R to the primary cilium, which maintained their signaling competence and regenerative ability [90]. Knockdown of IFT88 suppressed ciliogenesis in mouse 3T3-L1 preadipocytes and human mesenchymal stem cells (MSCs) through suppression of the IGF1R-Akt-PPARγ signaling pathway [91, 92].Further research uncovered the mechanism that elongated cilia prevent caveolin-1- and/or GM3-positive lipid rafts from being assembled around the ciliary base where insulin receptor proteins accumulate, thereby inhibiting insulin-Akt signaling [93]. In summary, the ciliary RTKs in various cell types can control important cellular and physiological processes context-dependent via the same downstream signaling pathways (e.g., the MAPK, PI3K-AKT, and phospholipase C γ pathway).

In summary, present evidence show the essential role of primary cilium in regulating various signaling pathways that initiated and controlled EMT program, suggesting the potential link between primary cilium and EMT. In the next section, we will list the direct evidence that shows the link between them.

## Direct evidence for ciliary signaling in EMT

During the study of primary cilium, mutations in ciliary proteins result in loss of cell polarity [67]—an EMT feature and inhibit the progress of endothelial-to-mesenchymal transition (EndoMT), a similar program like EMT [94]. In addition, ciliary proteins can disrupt the expression of SIX1 [95], which is implicated in EMT [96]. Meanwhile loss of IFT88 lead to increased SNAIL by activating the WNT/β-catenin signaling pathway, which consequently decreased expression of E-cadherin and increased expression of vimentin in β-cells [20]. Similarly, the epicardial cells in mice expressing a loss-of-function mutant form of Wdpcp, an essential component for primary ciliogenesis, showed defective ciliogenesis and reduced expression of EMT and mesenchymal markers, causing increased distribution of SMO into subcellular sites, where chemotactic signaling can be transduced and increased chemotactic response, thereby leading to faster progression of subepicardial plexus in Wdpcp mutant although it showed normal SHH signaling transcriptional responses [22]. Most recently researchers studied the activation of EMT, HH signaling pathway and the presence of primary cilium in normal and cancer tissues by immunohistochemical and ultrastructural techniques. They found a correlation between EMT beginning from urothelial basal cells and primary cilia assembly and suggest a potential implication of this structure in tumoral migration and invasiveness (likely in a Hh-dependent way) [25].

Conversely, initial cilium growth is followed by complete deciliation during epithelial–myofibroblast transition (EMyT), a more extreme type of EMT that can occur in kidney epithelial cells [97]. Furthermore, TGFβ-induced shortening of primary cilium was found in Madin Darby Canine Kidney (MDCK) cells. The deficiency of primary cilium caused by knocking down the ciliary proteins Arl13b and IFT20 exacerbated TGFβ-induced EMT, suggesting positive feedback between TGFβ release, shortening or distortion of the primary cilium, and enhanced EMT/fibrosis in the kidney [19]. The relationship was further supported by Wilson MM et al., they showed that EMT-TFs promote ciliogenesis upon entry into intermediate EMT states. The resulting primary cilium promote ubiquitination and inactivation of a transcriptional repressor(GLIS2) to promote MaSC stemness and induce the proliferative and tumorigenic capacities of the mammary tumor initiating cells (MaTICs) of claudin-low breast cancers [16, 24].

Although there is no direct evidence that cilium can affect EMT through NOTCH signaling. RBPjk, the essential DNA binding partner of NOTCH receptors, was required to regulate mesothelial EMT and select Clara versus ciliated cell fate in lung development. In this research the authors described a distal-to-proximal transition zone in which ciliated cells induce Notch activation in their neighbors, inhibiting them from selecting the same fate and permitting development of Clara cells [98], providing the indirect link between cilium-based NOTCH pathway with EMT. In a recent study, Multiple binding motifs associated with SNAIL-dependent transcriptional regulation were identified in close proximity to or within the FGFR1 promoter, a key inducer of ciliogenesis in the embryonic tissues of lower organisms, by reanalyzing the data from an existing SNAIL chromatin immuno-precipitation sequencing (ChIP-seq) study [24]. In summary these evidence verify the relationship between primary cilium and EMT.

## Conclusion

EMT is a remarkable mechanism that is essential for normal development, wound healing and carcinoma progression.

In the context of cancer, EMT and its reversed process, mesenchymal-to-epithelial transition (MET), confer additional malignant properties to cancer cells, including cancer stem cell activity [99] and more excellent resistance to chemotherapy and immunotherapy [100]. Previous research revealed that the presence or absence of the primary cilium could trigger or inhibit cancer progression depending on the cancer type and cancer-initiating mutations. The aberrant ciliogenesis could serve as a functional platform for a variety of cancer drug resistance mechanisms [101], suggesting that targeting primary cilium could be a promising treatment strategy. In addition, in the GBM and triple-negative breast cancer (TNBC) BAG3 knockout cells, enhanced ciliogenesis and reduced expression of SNAI1 and ZEB1 were correlated to decreased cell migration, suggesting that suppression of EMT and ciliogenesis as putative synergizing mechanisms of BAG3‐driven tumor aggressiveness in therapy‐resistant cancers [23].Understanding the ciliary signaling mechanisms that cooperate in the initiation and progression of EMT may lead to new therapeutic strategies to inhibit this cellular transformation in cancer. Nevertheless, limited studies were available to reveal the mechanism between primary cilium and EMT.

Primary cilium function as signal nexus to regulate signaling pathways that induce the initiation and progression of EMT, suggesting the potential link between primary cilium and EMT. Emerging evidence revealed the connection between EMT and primary cilium in multi organs and tissues, including epicardial tissue, kidney epithelial cells, pancreatic β-cells and the retinal pigment epithelium(RPE) [18–22] and in the context of cancer such as glioblastoma(GBM) and triple-negative breast cancer (TNBC) [23, 24], bladder cancer [25].But the relationship between primary cilium and EMT seem to be complicated and cell type, condition specific. For instance deficiency of primary cilium induces EMT in kidney epithelial cells [19, 97] and pancreatic β-cells [20], but inhibit EMT in the epicardial cells [22].These data prove that primary cilium function dual role upstream of EMT, but in the context of mammogenesis and claudin-low breast tumorigenesis, EMT programs induce primary ciliogenesis upon entry into intermediate transition states [24]. In addition, increased ciliogenesis caused by loss of BBS8 in RPE cells showed a EMT-like phenotype and the downstream EMT-TF SNAIL was only found in the nuclei of BBS8 mutant tissue [21].

In a word, emerging evidence has established the relationship between primary cilium and EMT under different conditions. Primary cilium interfere with EMT through signal transduction, but the mechanism is complex and puzzling and its details still need to be solved. The study of EMT axis of primary cilium has just begun and needs more effort.

## Data Availability

Not applicable.
